# Establishment of a Quantitative Method for the Extraction of Nicotine and Cotinine in Gingival Tissue and Relationship Between Gingival Intoxication With Conventional Smoking Biomarkers: A Pilot Study

**DOI:** 10.1002/cre2.70022

**Published:** 2024-12-17

**Authors:** Leila Salhi, Samuel Hazout, Dorien Van hede, France Lambert, Corinne Charlier, Marine Deville

**Affiliations:** ^1^ Department of Periodontology Oro‐Dental and Implant Surgery and Dental Biomaterials Research Unit University Hospital of Liège Liège Belgium; ^2^ Laboratory of Clinical, Forensic, Industrial and Environmental Toxicology University Hospital of Liège Liège Belgium; ^3^ Center for Interdisciplinary Research on Medicines CIRM research Unit University Hospital of Liège Liège Belgium

**Keywords:** cotinine extraction, cotinine tissue intoxication, gingival fibroblast, periodontitis

## Abstract

**Objectives:**

Smoking is considered a major risk factor for periodontitis genesis and progression. In clinical studies, specific indicators have been used to characterize the smoking status of the patient as the number of cigarettes consumed (NCC), the pack‐years (PY), or Fagerström Test for Nicotine Dependence (FTND). However, available literature is missing on the relationship between cotinine gingival intoxication and smoking indicators. First, the development of a quantitative method for the extraction of nicotine and cotinine in gingival tissue. Second, to investigate the relationship between gingival intoxication and conventional smoking biomarkers.

**Material and Methods:**

Fourteen smoker patients were included in the study. After clinical data collection, salivary and gingival samples collection, toxicological analyses were performed using liquid extraction after enzymatic digestion (subtilisin) and ultra‐performance liquid chromatography coupled to mass spectrometry (UPLC‐MS/MS).

**Results:**

Gingival cotinine quantification was successfully performed in 14 samples (100%) with a mean of 0.280 ng/mg (range = 0.094–0.505). Only FTND was statistically associated with gingival cotinine levels (*p* = 0.0072; *r*² = 0.60). Gingival nicotine quantification was achieved in 12 of the 14 gingival samples (86%) with a mean of 0.384 ± 1.00 ng/mg (range = 0.03–3.84). Gingival nicotine was statistically associated with NCC (*p* = 0.032; *r*² = 0.55), PY (*p* = 0.0011; *r*² = 0.76), and FTND (*p* = 0.016; *r*² = 0.60). Salivary nicotine and cotinine levels were statistically associated with, respectively, NCC (*p* = 0.030; *r*² = 0.34), and NCC (*p* = 0.0094; *r*² = 0.63) + PY (*p* = 0.0078; *r*² = 0.64).

**Conclusions:**

This pilot study established a quantitative extraction method for nicotine and cotinine from human gingival samples. Additionally, FTND was associated with gingival cotinine. However, further large‐scale studies are needed to confirm the relationship between nicotine dependence and gingival intoxication.

## Summary

- This study provides, first, a quantitative method for nicotine and cotinine extraction from human gingival samples.
- Second, the study highlights the association between nicotine/cotinine gingival tissue intoxication and conventional smoking indicators (pack‐year, number of cigarettes consumed, Fagerström Test for Nicotine Dependence).
- These results could be used for further studies related to tobacco and dentistry/periodontology, to select the accurate smoking indicators that reveal the gingival tissue intoxication responsible for oral diseases.

## 1 Introduction

Smoking is an addiction that impacts both oral and general health, with a high prevalence of oral diseases such as caries (Tomar et al. 2019), periodontitis (Tomar et al. 2019; Bergström and Preber 1994), and mucosal pathologies (Kansky et al. 2018). Moreover, this public health issue contributes to cancers (Johnson 2001), resulting in approximately eight million deaths annually worldwide (World Health Organization 2023). Among the cigarette components, nicotine remains the primary substance responsible for addiction (Balfour 2015). This addictive molecule quickly crosses the blood–brain barrier (Oldendorf 1992; Oldendorf, Stoller, and Harris 1993), interacting with areas of the cerebral cortex and the neurons of the mesolimbic dopaminergic neurons (Balfour 2015, 1994; Balfour et al. 2000), which are responsible for nicotine addiction trough their binding with nicotinic acetylcholine receptors (nAChRs) (Wonnacott, Sidhpura, and Balfour 2005). In addition to its distribution in the brain, nicotine is rapidly absorbed in several body tissues, showing a high affinity for the lungs, liver, kidneys, and spleen (Benowitz, Hukkanen, and Jacob 2009). Following absorption and distribution, 70%–80% percent of this molecule is metabolized in the liver through the cotinine pathway. Therefore, cotinine remains the main metabolite of nicotine and its most widely used biomarker, measurable in blood, urine, saliva, hair, or nails (Benowitz, Hukkanen, and Jacob 2009). Clinically, the degree of nicotine dependence can be evaluated with the Fagerström Test for Nicotine Dependence (FTND questionnaire) (Heatherton et al. 1991; Fagerstrom 2012). This indicator of smoking has been used to assess the association between smoking status and several general health diseases (Fagerstrom 2012; Payne et al. 1994) as well as periodontitis (Salhi et al. 2021, 2022; Han, Jeong, and Lee 2023).

Periodontitis is a chronic and inflammatory disease characterized by the presence of periodontal pathogens and the host's immune response against these specific bacteria (Kornman 2008).

Moreover, the disease is influenced by several risk factors, in particular smoking (Bergström, Eliasson, and Dock 2000). A recent systematic review found that smoking was associated with a higher prevalence of periodontitis, with an odds ratio (OR) of 2.78 (Aminoshariae, Kulild, and Gutmann 2020). Furthermore, a recent meta‐analysis confirmed that smoking adversely affects the incidence and progression of periodontitis, with a risk ratio (RR) of 1.85 (Leite et al. 2018, 2019).

The clinical studies on the relationship between smoking and periodontitis have classified individuals as smokers and nonsmokers or have used various conventional smoking indicators, such as the number of cigarettes consumed daily (NCC) (Payne et al. 1994; Salhi et al. 2020) or the pack‐years (PY) (Costa and Cota 2019; Nishida et al. 2005). However, these indicators do not accurately reflect the gingival cotinine intoxication associated with the severity of periodontitis, as demonstrated by salivary (Chen et al. 2001) and plasma (Leow et al. 2006; Xu et al. 2002) biomarkers. In vitro studies have assessed the impact of nicotine and cotinine on human gingival fibroblasts (HGFs), finding that nicotine and its byproducts inhibit proliferation, cell adhesion, and migration of these cells (Tatsumi et al. 2021; Tipton and Dabbous 1995). Therefore, the primary aim of this pilot study was to develop a method for extracting nicotine and cotinine from human gingival samples. The secondary aim was to investigate the relationship between conventional smoking indicators and gingival nicotine/cotinine intoxication.

## 2 Materials and Methods

### 2.1 Ethical Approval

The study protocol received approval from the Ethics Committee of the University Hospital, University of Liege, Belgium (2021/250), and was conducted in accordance with the Helsinki Declaration of 1975, as amended in 2013. The objectives and procedures of the study were thoroughly explained to all participants, who then provided written informed consent. Furthermore, the study was registered on clinicaltrial.gov (file number: NCT05736250).

### 2.2 Study Population

The study population included 14 smoking patients undergoing dental treatments requiring surgical intervention under local anesthesia at the Department of Periodontology and Oral Surgery of the University Hospital, Liege, Belgium.

Participants met the following inclusion criteria: (1) current smokers, (2) aged 18 years or older, and (3) in need of a surgical procedure under local anesthesia (e.g., extraction, periodontal surgery, scaling, and root planning). Exclusion criteria comprised the following: (1) diabetes, (2) connective tissue disease, (3) pregnancy, (4) undergoing radiotherapy, (5) undergoing chemotherapy, and (6) presence of psychological disorders.

### 2.3 Smoking Status

Smoking status was determined using three methods: (1) the NCC, (2) the PY, and (3) the FTND. The NCC was assessed by asking the patient “How many cigarettes do you smoke per day?.” To calculate the PY, the patients were asked “How long they had been smoking?” and the ratio of the NCC to smoking years gives the PY score. The FTND included six questions with the following scoring system: (1) time until first cigarette after waking (within 5 min: 3 points; 6–30 min: 2 points, 31–60 min: 1 point; after 60 min: 0 point); (2) difficulty refraining from smoking in forbidden places (yes: 1 point; no: 0 point); (3) cigarette hardest to give up (the first in the morning: 1 point; another: 0 point); (4) daily cigarette count (10 or fewer: 0 point; 11–20: 1 point; 21–30: 2 points; 31 or more: 3 points); (5) increased morning smoking frequency (yes: 1 point; no :0 point); and (6) smoking while ill in bed (yes: 1 point; no: 0 point). The total FTND score, summing all six responses scores, ranges from 0 to 10, with the higher scores indicating stronger smoking dependence (Fagerstrom 2012).

### 2.4 Data Collection

#### 2.4.1 Salivary Samples Collection

One hour after smoking the last cigarette, the unstimulated saliva (1 mL) was collected using a sterile, single‐use pipette and stored in a sterile tube at −20°C until analysis.

#### 2.4.2 Gingival Samples Collection

Gingival connective tissue was harvested during dental/periodontal surgeries. Collected gingival biopsies were stored in a sterile tube at −20°C until analysis.

### 2.5 Toxicological Analyses

All toxicological analyses were carried out in the Clinical, Forensic, Environmental, and Industrial Toxicology Laboratory of the University Hospital, University of Liege, Belgium.

Following enzymatic digestion of gingival samples with subtilisin, salivary and gingival specimens underwent liquid–liquid extraction. The resulting extracts were analyzed using ultra‐performance liquid chromatography coupled with mass spectrometry (UPLC‐MS).

#### 2.5.1 Supplies and Reagents

Certified reference standards of cotinine and cotinine‐d3 (used as internal standard) were purchased from LGC standards (Teddington, UK), while nicotine and nicotine‐d4 were obtained from Merck (Darmstadt, Germany), along with Na_2_CO_3_, subtilisin A (Protease from *Bacillus licheniformis*) and TRIS (Tris(hydroxymethyl)aminomethane). All solvents were of LC‐MS or HPLC grade and supplied by J.T. Baker (Phillipsburg, USA). Ammonium formate for mobile phase preparation was acquired from Fisher Chemical (Merelbeke, Belgium).

#### 2.5.2 Treatment of Gingival Samples

Once defrosted, gingival samples were weighed for quantity adjustments based on the sample weight. The samples were placed in a tube containing 150 µL of subtilisin solution (10 mg dissolved in 15 mL of TRIS buffer 1 M pH 7.4) and 100 µL of water, stirred for 45 min, heated in an oven at 56°C for 1 h, and centrifuged at 3000 rpm for 10 min. The samples were then ready for the liquid phase extraction process.

#### 2.5.3 Extraction Technique

After adding the internal standard solution (nicotine‐d4 and cotinine‐d3, 0.5 mg/L) and the adjustment of pH with Na_2_CO_3_ (1 M), 0.5 mL of a mixture comprising diethyl ether, dichloromethane, *n*‐hexane, and *n*‐amylic alcohol in a ratio of 50/30/20/0.5 (*V*/*V*) was added to 100 µL of the sample. The samples were vortexed for 1 min and then centrifuged at 10,900 rpm for 5 min. The organic layer was evaporated under nitrogen at a temperature not exceeding 40°C and reconstituted in 100 µL of mobile phase for subsequent chromatographic analysis.

#### 2.5.4 Calibration Curve

Four working solutions were prepared to achieve nicotine and cotinine concentrations of 10, 1, 0.1, and 0.01 mg/L by dissolving the substances in methanol and performing successive dilutions. Ten calibration levels were prepared by spiking water with an appropriate volume of working solution, resulting in final concentrations of 0.5, 1.0, 2.0, 5.0, 10.0, 50.0, 100, 500, 1000, and 2500 ng/mL for both nicotine and cotinine. Quality control samples (QCs) were prepared in a similar manner, using an independent stock solution.

#### 2.5.5 Instrumental Analysis

Post‐extraction, the biological specimens were analyzed by Ultra‐High Performance Liquid Chromatography (UHPLC‐MS/MS). The UHPLC‐MS/MS system was an Acquity UPLC I‐Class coupled to a Xevo TQ‐S, equipped with an electrospray ion (ESI) source, both from Waters (Milford, USA). The signal acquisition and peak integration were performed using the MassLynx v4.1 software suite supplied by Waters.

Ten microliters of the extract were injected into an Acquity UPLC BEH C18 column (2.1 × 50 mm, 1.7 μm) from Waters. The column oven temperature was controlled at 45°C. The mobile phase, consisting of ammonium formate (A) and acetonitrile (B), was delivered at a flow rate of 0.3 mL/min. Gradient elution was programmed as follows: starting conditions 5% B; maintained during 30 s, increasing to 30% B between 0.5 and 4 min; further increase to 95% B between 4 and 4.5 min. This composition was kept for 0.5 min and finally returned to the initial conditions by 6.00 min for 1.5 min re‐equilibration.

The mass spectrometer operated in positive mode (1 kV) with desolvation achieved by a nitrogen flow of 800 L/h at 450°C. The cone gas (nitrogen) and collision gas (argon) flow rates were set at rates of 150 L/h and 0.2 mL/min, respectively. The multiple reaction monitoring parameters are detailed in the Supporting Information.

### 2.6 Statistical Analysis

Results are expressed as means and standard deviations (SDs), quartiles (median, Q1, Q3), and extremes (minimum, maximum) for quantitative variables. Logarithmic transformation was applied to normalize the distribution of certain variables. The Pearson's correlation coefficient was used to evaluate the relationship between two continuous variables. Comparisons of mean values between the two groups were conducted using the Student's *t*‐test. Both univariate and multivariate regression models were employed to examine the relationship between a continuous variable and one or more covariates. The parameter estimate (*β*), along with its standard error (SE) and *p*‐value, is reported. The coefficient of determination (*r*²) indicates the proportion of the total variability in the dependent variable (*Y*) that is explained by the covariates. A value closer to 1 denotes a stronger model fit. Results were considered statistically significant at a 5% level of uncertainty (*p* < 0.05). Computations were conducted using SAS version 9.4, while graphical illustrations were generated using R version 4.2.2.

## 3 Results

### 3.1 Patient Characteristics and Conventional Smoking Indicators

No significant difference was observed between genders (male and female) in terms of tobacco consumption.

### 3.2 Nicotine and Cotinine Quantification in Oral Samples

The extraction technique and the molecule quantification were successfully achieved for each sample (Supporting Information).

Mean salivary nicotine was 856 ng/mL ± 965 with no statistical difference between genders (*p* = 0.34).

Mean salivary cotinine was 339 ng/mL ± 136 with no statistical difference between genders (*p* = 0.08).

Mean gingival nicotine was 0.384 ng/mg ± 1.00 with no statistical difference between genders (*p* = 0.16).

Mean gingival cotinine was 0.28 ng/mg ± 0.13 with no statistical difference between genders (*p* = 0.23).

### 3.3 Association Between Salivary Cotinine and Smoking Indicators

**Figure 1:**
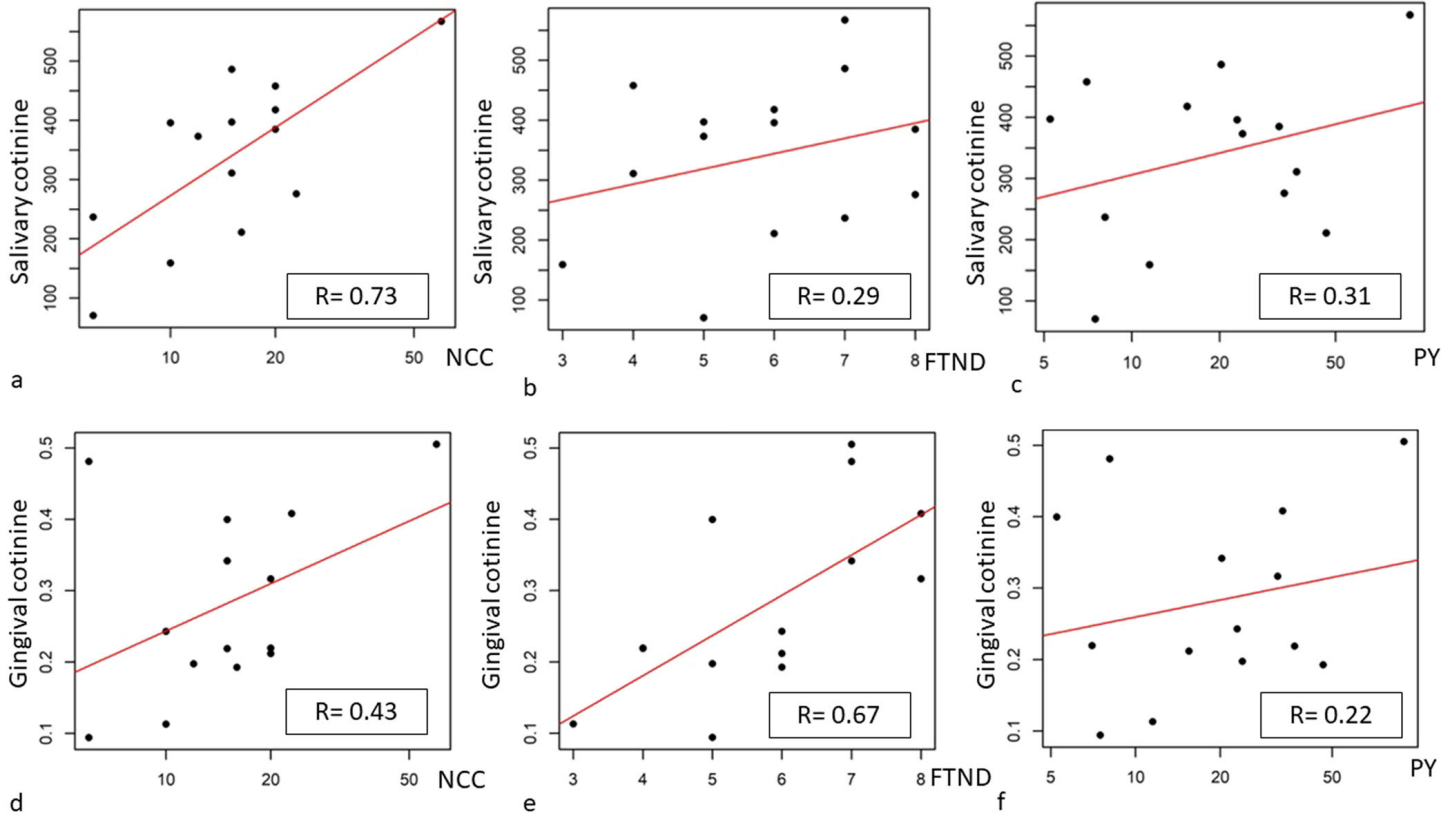
Association between cotinine in oral samples and smoking indicators. F1a, association between salivary cotinine (ng/ml) and NCC; F1b, association between salivary cotinine (ng/mL) and FTND; F1c, association between salivary cotinine (ng/mL) and PY; F1d, association between gingival cotinine (ng/mg) and NCC; F1e, association between gingival cotinine (ng/mg) and FTND; F1f, association between gingival cotinine (ng/mg) and PY.

At the univariate analysis level, a significant association was found between salivary cotinine and the NCC (*p* = 0.0043). After adjusting for age and gender, salivary cotinine showed a significant association with both NCC (*p* = 0.0094) and PY (*p* = 0.0078) (Table 1, Figure 1).

**Table 1:** Association of salivary cotinine/nicotine and smoking indicators.

Parameters	*N*	*β* (SE)	*p*‐value	*r*²
Salivary cotinine				
*Models adjusted for age and gender*				
Model with NCC	14			**0.63**
Gender (male)		−73.0 (54.9)	0.21	
Age (years)		−1.52 (1.89)	0.44	
NCC (Ln)		148 (46.3)	0.0094*	
Model with FTND	14			0.33
Gender (male)		−108 (72.3)	0.17	
Age (years)		−1.71 (2.58)	0.52	
FTND		24.4 (23.3)	0.32	
Model with PY	14			**0.64**
Gender (male)		−90.0 (52.9)	0.12	
Age (years)		−7.15 (2.54)	0.019*	
PY (Ln)		142 (42.7)	0.0078*	

Salivary nicotine				
*Models adjusted for age and gender*				
Model with NCC	14			0.42
Gender (male)		−0.50 (0.60)	0.42	
Age (years)		0.021 (0.021)	0.34	
NCC (Ln)		1.09 (0.50)	0.056	
Model with FTND	14			0.22
Gender (male)		−0.76 (0.67)	0.29	
Age (years)		0.019 (0.024)	0.45	
FTND		0.22 (0.22)	0.33	
Model with PY	14			0.39
Gender (male)		−0.64 (0.59)	0.31	
Age (years)		−0.018 (0.029)	0.54	
PY (Ln)		0.98 (0.48)	0.069	

*Note:* Significant *p*‐values (*p* < 0.05) are marked with *and p‐values with a weak evidence (*p* < 0.1) are underlined. Bold r2 values indicate moderate associations.

Abbreviations: FTND, Fagerström Test for Nicotine Dependence; NCC, number cigarettes consumed; PY, pack‐years.

### 3.4 Association Between Salivary Nicotine and Smoking Indicators

At the univariate analysis level, salivary nicotine demonstrated a significant association with NCC (*p* = 0.030). However, after adjusting for age and gender, only a trend for NCC and PY was observed (Table 1).

### 3.5 Association Between Gingival Nicotine and Smoking Indicators

Gingival nicotine (considering “traces” = 0.03 n/mg) was significantly associated with FTND (*p* = 0.014), NCC (*p* = 0.018), and PY (*p* = 0.0056) at the univariate level and remained significant after adjustment for age and gender. The model including PY as a variable showed the best fit based on the *r*² (Table 2).

**Table 2:** Association of gingival cotinine/nicotine and smoking indicators.

Parameters	*N*	*β* (SE)	*p*‐value	*r*²
Gingival cotinine				
*Models adjusted for age and gender*				
Model with NCC	14			0.28
Gender (male)		−0.048 (0.073)	0.52	
Age (years)		−0.0018 (0.0025)	0.50	
NCC (Ln)		0.083 (0.061)	0.21	
Model with FTND	14			**0.60**
Gender (male)		−0.054 (0.053)	0.33	
Age (years)		−0.0025 (0.0019)	0.22	
FTND		0.057 (0.017)	0.0072*	
Model with PY	14			0.42
Gender (male)		−0.052 (0.064)	0.44	
Age (years)		−0.0062 (0.0031)	0.071	
PY (Ln)		0.11 (0.052)	0.057	

**Gingival nicotine**				
*Models adjusted for age and gender*				
Model with NCC	14			**0.55**
Gender (male)		−0.96 (0.60)	0.14	
Age (years)		0.032 (0.021)	0.16	
NCC (Ln)		1.27 (0.51)	0.032*	
Model with FTND	14			**0.60**
Gender (male)		−1.16 (0.55)	0.062	
Age (years)		0.026 (0.020)	0.22	
FTND		0.51 (0.18)	0.016*	
Model with PY	14			0.76*
Gender (male)		−1.04 (0.43)	0.037*	
Age (years)		−0.031 (0.021)	0.16	
PY (Ln)		1.57 (0.35)	0.0011*	

*Note:* Significant *p*‐values (*p* < 0.05) are marked with * and *p*‐values with a weak evidence (*p* < 0.1) are underlined. Bold r2 values indicate moderate associations.

Abbreviations: FTND, Fagerström Test for Nicotine Dependence; NCC, number cigarettes consumed; PY, pack‐years.

### 3.6 Association Between Gingival Cotinine and Smoking Indicators

Gingival cotinine demonstrated a significant association with the FTND score both in the univariate analysis level (*p* = 0.0091) and after adjustments for age and gender (*p* = 0.0072) (Table 2, Figure 1).

### 3.7 Association Between Toxicological Indicators

No toxicological parameter is associated with age or gender. Among the toxicological indicators, gingival cotinine was positively associated with nicotine in saliva (*r* ^2^ = 0.61, *p* = 0.019) as well as in gingival tissue (*r* ^2^ = 0.64, *p* = 0.014). Nicotine and cotinine association in oral samples are summarized in Table 3.

**Table 3:** Nicotine and cotinine association in oral samples.

Pearson's correlation coefficients						
Prob > |*r*| under H0: *ρ* = 0						
Number of observations						
	Age	Salivary nicotine (Ln)	Tissulary nicotine (0) (Ln)	Tissulary nicotine (0.03) (Ln)	Salivary cotinine	Tissular cotinine
Age		0.16	0.37	0.20	−0.28	−0.27
		0.58	0.24	0.49	0.34	0.34
		14	12	14	14	14
Salivary nicotine (Ln)			0.43	0.45	**0.61**	0.27
			0.16	0.11	0.019*	0.35
			12	14	14	14
Tissulary nicotine (0) (Ln)				100.000	0.34	**0.59**
				< 0.0001*	0.28	0.046*
				12	12	12
Tissulary nicotine (0.03) (Ln)					0.42	**0.64**
					0.14	0.014*
					14	14
Salivary cotinine						**0.51**
						0.064
						14
Tissular cotinine						

*Note:* Significant *p*‐values (*p *< 0.05) are marked with * and *p*‐values with weak significance (*p* < 0.1) are underlined. Bold r values indicate moderate associations.

## 4 Discussion

This pilot study primarily established a quantitative method for extracting nicotine and cotinine from gingival tissue. Furthermore, a significant association between gingival cotinine levels and the FTND was found, suggesting a systemic effect of nicotine leading to dependence.

The quantification protocol employed leveraged well‐established digestion methods used for other biological tissues such as the liver, kidney, and skin (Clark, Zhang, and Anderson 2016). Upon dissolution of the gingival tissues, a conventional LC‐MS method was applied for component quantification (Baumann et al. 2010; Shakleya and Huestis 2009; Yue et al. 2010). However, the initial phase of this study encountered a notable challenge with trace quantities within samples from two moderate smokers of the 14 included. During the first three attempts of gingival nicotine analysis, the detected levels were exceptionally low, primarily registering as trace quantities within samples from two moderate smokers. Interestingly, among these preliminary analyses, the presence of nicotine was unequivocally confirmed in just one instance, coinciding with a heavy smoker patient who consumed 60 cigarettes per day. To address this sensitivity issue, transitions were checked and another mass spectrometer was used. This yielded promising outcomes. Subsequently, this second approach enabled the accurate quantification of gingival nicotine within the remaining 11 samples. Nonetheless, the quantified tissue nicotine values remained relatively low, underscoring the technique's sensitivity in dosing this biomarker. This relatively low level of nicotine detected in gingival tissue can be explained by the pronounced metabolism of nicotine into its primary metabolite: cotinine. Indeed, nicotine has a short half‐life (2 h), whereas cotinine has the advantage of a fairly long half‐life (16 h). This is why cotinine is most frequently used as a biomarker for tobacco smoke in biological samples (Benowitz 1996), and it should not be different for gingival specimens.

Previous studies indicate that only a small fraction of nicotine from smoke is absorbed buccally, even when it is held in the mouth for a long time, which could explain the remanence of nicotine in saliva (Gori, Benowitz, and Lynch 1986). Therefore, gingival cotinine levels more accurately reflect systemic exposure, while salivary levels indicate a more transient exposure. This pilot study supports the hypothesis that gingival cotinine is closely related to systemic nicotine effects and dependence, whereas salivary nicotine and cotinine levels correlate with NCC and PY.

These findings underscore the importance of interpreting smoking indicators contextually. Indeed, the nicotine dependence reflect the systemic effect of nicotine and its “in situ‐effect” on periodontal tissues (Salhi et al. 2021), through the cotinine levels in gingiva, contributing to periodontitis severity. The gingival cotinine intoxication may explain the detrimental effects on cellular behavior involved in the disease genesis and progression (Tatsumi et al. 2021; Tipton and Dabbous 1995), Indeed, in vitro studies supported that smoking alters or suppresses cellular functions of HGFs, such as their migration (Torshabi et al. 2017), their growth, and their production of collagen (Tipton and Dabbous 1995), accelerating the genesis and progression of periodontal diseases. Therefore, the FTND and the gingival cotinine intoxication seem to represent a more relevant indicator to assess smoking status than the PY and salivary nicotine in the context of periodontitis.

However, as PY and its associated NCC reflect the degree of exposure to carcinogenic substances, such as polycyclic aromatic hydrocarbons and tobacco‐specific nitrosamines (Aredo et al. 2021; Pankow et al. 2007), these smoking indicators stand as valuable tools for assessing overall tobacco exposure and carcinogenesis. Indeed, even after smoking cessation, the PY will be unchanged., Therefore, PY and NCC represent relevant indicator in studies related oncology, instead of periodontology.

Even if this study does not allow us to conclude the potential association between gingival cotinine, FTND, and periodontitis, the available literature supports this relationship. Notably, a cross‐sectional study conducted on 800 subjects, which assessed patient status using the FTND, found an association between higher FTND scores and worse periodontal health (Goyal et al. 2019). Furthermore, other recent studies have also concluded that periodontitis severity was associated with FTND scores (Salhi et al. 2021, 2022). This pilot study faced limitations, including a small sample size and the localization of tissue harvesting. To more conclusively determine, the relationship between gingival exposure to tobacco, smoking indicators, and periodontal health, further research with larger sample sizes is essential.

## 5 Conclusion

This pilot study has successfully established a quantitative extraction method for nicotine and cotinine from human gingival samples. Additionally, FTND was associated with gingival cotinine. However, further large‐scale studies are needed to confirm the relation between nicotine dependence and gingival intoxication.

## Author Contributions

**Leila Salhi:** conceptualization, methodology, validation, investigation, resources, writing–original draft preparation, writing–review and editing, visualization. **Samuel Hazout:** resources, writing–original draft preparation. **Dorien Van hede:** validation, writing–original draft preparation, writing–review and editing. **France Lambert:** validation. **Corinne Charlier:** validation. **Marine Deville:** methodology, validation, writing–original draft preparation, writing–review and editing, visualization. All authors have read and agreed to the published version of the manuscript.

## Conflicts of Interest

The authors declare no conflicts of interest.

## Supporting information

Supporting information.

## Data Availability

The data supporting this manuscript is available from the corresponding author upon reasonable request.
