# Supplementary material for: Establishment of a Quantitative Method for the Extraction of Nicotine and Cotinine in Gingival Tissue and Relationship Between Gingival Intoxication With Conventional Smoking Biomarkers: A Pilot Study
Source: Clin Exp Dent Res. 2024 Dec 17;10(6):e70022. doi: 10.1002/cre2.70022 (PMC11650890; doi:10.1002/cre2.70022)
Supplement: Supplementary file 1 — Supporting information. [file CRE2-10-e70022-s001.docx]

SUPPLEMENTARY MATERIAL

Appendix 1. The multiple reaction monitoring parameters, cone voltages and collision energies for nicotine, cotinine, and their respective internal standards

|  | m/z precursor | m/z product | cone voltage | collision energy |  |
| --- | --- | --- | --- | --- | --- |
|  | ion | ion | (v) | (eV) |  |
| Nicotine | 162.8 | 116.7 | 22 | 25 |  |
| Nicotine | 162.8 | 129.7 | 22 | 30 |  |
| Nicotine-d4 | 166.8 | 120.7 | 25 | 25 |  |
| Cotinine | 176.8 | 79.7 | 30 | 25 |  |
| Cotinine | 176.8 | 97.7 | 30 | 25 |  |
| Cotinine-d3 | 179.8 | 79.7 | 30 | 25 |  |

m: mass, z: charge number of ions, v:volt, eV:electronvolt, d-deuterated

| appendix. 2 Nicotine and cotinine concentrations in oral samples. | | | |
| --- | --- | --- | --- |
| Variable | N | Mean± SD (ng/mg) | P-value |
|  |  |  |  |
| Salivary nicotine |  | 856 ± 965 |  |
| Female | 8 | 1125 ± 1164 | 0.34* |
| Male | 6 | 499 ± 505 |  |
| Gingival nicotine |  | 0.384 ± 1.00 |  |
| Female | 8 | 0.61 ± 1.31 | 0.16* |
| Male | 6 | 0.09 ± 0.08 |  |
| Salivary cotinine |  | 339 ± 136 |  |
| Female | 8 | 393 ± 121 | 0.08* |
| Male | 6 | 266 ± 129 |  |
| Gingival cotinine |  | 0.28 ± 0.13 |  |
| Female | 8 | 0.32 ± 0.13 | 0.23* |
| Male | 6 | 0.23 ± 0.12 |  |

*testing log
